# Serum Vitamin D Levels in Pediatric Tuberculosis Patients in a Tertiary Care Center in India: A Case-Control Study

**DOI:** 10.7759/cureus.39937

**Published:** 2023-06-04

**Authors:** Karthik Ageeru, Suresh Babu Mendu, Santhosh Avinash, Srinivasa Kalyani, Rakesh Kotha

**Affiliations:** 1 Pediatrics, Niloufer Hospital, Hyderabad, IND; 2 Pediatrics, Government Medical College Siddipet, Siddipet, IND; 3 Pediatrics, Osmania Medical College, Hyderabad, IND; 4 Neonatology, Osmania Medical College, Hyderabad, IND

**Keywords:** children, vitamin d deficiency, mycobacterium, malnutrition, cathelicidin

## Abstract

Background

*Mycobacterium* is certainly one individual organism contributing to the most deaths of children among the world’s lower- and medium-income nations. According to earlier studies, vitamin D insufficiency is one of the risk factors. We undertook this study since very few case-control studies are present. This study aimed to evaluate the role of vitamin D in tuberculosis (TB).

Methods

This case-control study was carried out in a tertiary care center at Niloufer Hospital over a period of one year and five months. The sample size was 140. SPSS (Statistical Package for the Social Sciences) Version 19 (IBM Corp., Armonk, NY) was used for statistical analysis. Two-tailed p-values and odds ratios were obtained. The chi-square test was applied to differentiate between two categorical variables. For means, the Student t-test was applied. We usually take baseline investigations before starting ATT (anti-TB treatment) with the blood sample we tested for vitamin D levels.

Results

With p-values of 0.767 and 0.866, the age and sex distributions in the cases and controls were comparable. Rural and urban area distribution and malnutrition distribution were not similar in both groups, with a p-value of 0.001. The mean vitamin D level in cases was 10.4, while controls it was 22.8, and this difference is statistically significant (p = 0.001).

Conclusion

Vitamin D deficiency is more common in children with TB than in normal children. In addition, a severe form of vitamin D deficiency (less than 10 ng/mL) was higher among children with TB. Clinicians should be aware of associated malnutrition and low socioeconomic status as risk factors for severe vitamin D deficiencies among them.

## Introduction

Tuberculosis (TB) is one of the most devastating and widespread infections in the world. It is a leading cause of morbidity and mortality, particularly in developing countries. It should also be noted that children, particularly older children and adolescents, can be infected. TB is caused by mycobacteria. *Mycobacterium tuberculosis *is the most frequently found organism, and to a lesser extent, so are *M. bovis* and *M. africanum*. An imbalance between mycobacterial virulence and host immunity determines the progress of an infection/disease [1,2].

Factors related to the risk of TB include low socioeconomic status, poor nutrition, traditional or cultural traits, tobacco smoking, contact with sputum smear-positive index patients, and genetic susceptibility, one of which is vitamin D deficiency (VDD). Studies have found that 1,25(OH)2D3 binds to the vitamin D receptor (VDR), activates VDR signalling, and induces a series of antimicrobial responses such as induction of autophagy and phagolysosomal fusion, release and activation of the antimicrobial peptide cathelicidin, and killing of intracellular *Mycobacterium tuberculosis* [2]. Vitamin D inhibits bacterial growth by inducing LL-37/hCAP-18 (cathelicidin), the microbial peptide CAMP (collection of anti-microbial peptides), and autophagic flux in macrophages infected with *Mycobacterium tuberculosis* bacilli. The methylation levels of the VDR genes in the metabolic pathway of vitamin D are related to the risk and prognosis of TB [3]. Many studies have shown a positive correlation between low levels of serum vitamin D (<20 ng/mL) and TB. Though there is little evidence regarding the range of VDD in different types of TB and its severity, there are very few pediatric case-control studies available in the literature. The goal of our study is to fill this gap. As such, we undertook this study to determine the function of hypovitaminosis D as a risk factor for TB by evaluating serum levels and comparing serum vitamin D levels in healthy children.

## Materials and methods

This was a prospective case-control study conducted at Niloufer Hospital for Women and Children in Hyderabad, Telangana, India. Our study was done over a period of one year and five months (January 2019 to June 2020). Children between 6 months and 12 years of age who had been recently diagnosed or were already on anti-tuberculous (clinical, radiological, or laboratory-proven diagnosis) treatment as cases were included. Normal children between 6 months and 12 years of age comprised the study controls, excluding children younger than 6 months and greater than 12 years, children with acute respiratory tract infections or other comorbidities, children with previous VDD and on vitamin D supplementation, and those who did not give consent.

Data entry was conducted using the Microsoft Excel 2018 version, while data analysis was conducted using SPSS (Statistical Package for the Social Sciences) Version 19 (IBM Corp., Armonk, NY). The data were presented as percentages. The chi-square test was used to investigate the relationship between variables. Odds ratios and two-tailed p-values were computed. A two-tailed p-value of less than 0.05 is considered statistically significant. The sample size was calculated at 70 in each group based on the results of the existing literature [4]. We analyzed the data at 80% power and 0.05 type I error with a 95% confidence interval. The sample size was calculated from the following source: https://select-statistics.co.uk/calculators/sample-size-calculator-population-proportion. We received ethical committee approval with the number ECR/300/inst2013/AP/RR-16. Informed consent was obtained from the entire study population. Information was collected from patients by a trained research doctor who was not involved in the study using a structured proforma. Patients with TB were matched to children in the control population in terms of age, sex, demographic distribution, BCG (bacille Calmette-Guerin) vaccination, malnutrition, and socioeconomic status. We hypothesized that similar results would be found in children. We performed a prospective case-control study using a study population of 140 with 70 cases and controls each. Our institute population is representative of the current pediatric population of Hyderabad. Cases and controls were compared using the chi-square test and paired t-test to exclude possible confounding factors, and the results were adjusted accordingly. We usually take baseline investigations before starting ATT (anti-TB treatment) with the blood sample we tested for vitamin D levels. We obtained informed written consent. As such, we undertook this study to determine the function of hypovitaminosis D as a risk factor for TB by evaluating serum levels and comparing serum vitamin D levels in healthy children.

## Results

A total of 70 children with TB between the ages of 6 months and 12 years were included in our study. The study population was divided into three groups according to age: 1-4 years, 5-8 years, and 9-12 years. In cases, 24 (34.3%), 26 (37.1%), and 20 (28.6%) were present, and in controls, 22 (31.4%), 24 (34.3%), and 24 (34.3%) were present in the respected age groups. Both groups were statistically similar, with a p-value of 0.767 and a chi-square value of 0.531. In these cases, there were 36 (51.4%) females and 34 (48.6%) males. In controls, there were 35 (50%) females and 35 (50%) males. Both groups were statistically similar with a p-value of 0.866 and a chi-square value of 0.029. Both urban and rural groups were statistically not similar in terms of demographics, with a p-value of 0.001 and a chi-square value of 16.943. In these cases, 12 (17.1%) of the children were from rural areas, while 58 (82.9%) were from metropolitan areas. In the controls, 35 (50%) of the children were from rural areas, and another 35 (50%) were from urban areas.

In cases of mild, moderate, and severe malnutrition, there were 7 (10%), 22 (31.4%), and 35 (50%) children; in controls, it was 7 (10%), 4 (5.7%), and 0 (0%) (Table 1), which were statistically not similar.

**Table 1:** Different levels of malnutrition in both cases and controls Chi-square value = 90.677, p-value = 0.001

			Group		Total
			Case	Control	
Malnutrition	Mild	Count	7	7	14
		%	10.0%	10.0%	10.0%
	Moderate	Count	22	4	26
		%	31.4%	5.7%	18.6%
	Severe	Count	35	0	35
		%	50.0%		25.0%
	No	Count	6	59	65
		%	8.6%	84.3%	46.4%
Total		Count	70	70	140
		%	100.0%	100.0%	100.0%

In the case group, in terms of socioeconomic classes, classes 2, 3, and 4, accounted for three (4.3%), 33 (47.1%), and 34 (48.6%) cases, respectively; in the control group, they accounted for 8 (11.4%), 32 (45.7%), and 30 (42.9%) cases, respectively. Both groups were statistically similar in terms of their socioeconomic status, with a p-value of 0.281 and a chi-square value of 2.538. BCG vaccination was taken in 60 (85.7%) cases in the case group and 67 (95.7%) cases in the control group. Both groups were statistically not similar with a p-value of 0.042 and a chi-square value of 4.155 (Table 2). TB contact was present in six (8.6%) cases in both controls and cases.

**Table 2:** BCG vaccination status in cases and controls Chi-square value = 4.155, p-value = 0.042 BCG, bacille Calmette-Guerin

			Group		Total
			Case	Control	
BCG	No	Count	10	3	13
		%	14.3%	4.3%	9.3%
	Yes	Count	60	67	127
		%	85.7%	95.7%	90.7%
Total		Count	70	70	140
		%	100.0%	100.0%	100.0%

Serum vitamin D levels are classified into three categories: 10 ng/mL, 10-20 ng/mL, and more than 20 ng/mL. In the category of less than 10 ng/mL, there were 52 (74.3%) cases and 15 (21.4%) controls. In the category of 10-20 ng/mL, there were 7 (10%) cases and 17 (24.3%) controls. In the category of more than 20 ng/mL, there were 11 (15.7%) cases and 38 (54.3%) controls. A p-value of 0.001 indicates that statistically significant difference was present in both groups (Table 3).

**Table 3:** Serum vitamin D levels in cases and controls Chi-square value = 39.477, p-value = 0.001, odds ratio = 6.190

Variable			Group		Total
			Case	Control	
Vitamin D group (ng/mL)	<10	Count	52	15	67
		%	74.3%	21.4%	47.9%
	10-20	Count	7	17	24
		%	10.0%	24.3%	17.1%
	>20	Count	11	38	49
		%	15.7%	54.3%	35.0%
Total		Count	70	70	140
		%	100.0%	100.0%	100.0%

The mean vitamin D level in the case group was 10.4307 ng/mL with a standard deviation of 6.74755, and in the control group, it was 22.8449 ng/mL with a standard deviation of 12.13584 (Table 4).

**Table 4:** Mean age and mean vitamin D levels in cases and controls

	Group	N	Mean (ng/mL)	Standard Deviation	P-value
Age	Case	70	6.09	3.243	0.323
	Control	70	6.64	3.401	
Vitamin D levels	Case	70	10.4307	6.74755	0.001
	Control	70	22.8449	12.13584	

## Discussion

In our institute during 2018-2019, 302 TB cases were present. Most of them were male and of low socioeconomic status. The mean vitamin D level in our study was 10.43 ng/ml (±6.74 ng/mL standard deviation) among the cases and 22.84 ng/mL (±12.13 ng/mL standard deviation) among the controls; the difference was statistically significant with a p-value of 0.001. The incidence of TB among patients with low vitamin D (both insufficiency and deficiency) was 6.19 (an OR of 6.1904). Among the cases, 85.15% had low vitamin D (insufficiency and deficiency) (n = 59) compared to 65% (n = 32) in controls, which was statistically significant (p = 0.001). Similar results were obtained in various other studies, such as the one by Talat et al., in which 79% of the cases had VDD and 58% of the controls had VDD (p = 0.003) [5]. A systematic review by Sutaria et al. reported that patients with TB have lower serum vitamin D than healthy controls [6], but in Yani et al.'s study, the p-value was 0.32, which is statistically insignificant [7]. Previous studies also indicate that a low serum vitamin D level is associated with a higher risk of developing TB. Arnedo-Pena et al. reported that vitamin D sufficiency protects adults with a history of close TB contact against TB [8]. Furthermore, a study conducted by Talat et al. investigated the role of vitamin D in TB progression and revealed that low serum vitamin D levels were associated with a five-fold increased risk for progression to TB. Ho-Pham reported that vitamin D insufficiency is a risk factor for TB in adult men [9]. A Japanese study conducted by Sato et al. suggested that low serum 25-hydroxyvitamin D levels may not only increase the risk of developing active TB but may also be related to poor treatment outcomes [10].

In our study, the mean age in cases was 6.1 years (SD: 3.24 years) and the mean age in controls was 6.6 years (SD: 3.40 years) in comparison to studies carried out by Venturini et al. and Buonsenso et al. [11,12]. In Shah and Chilkar's study, the mean age was less, with 4.1 years and 4.8 years in cases and controls, respectively [13]. Talat et al. reported mean ages to be 10.3 and 11.2 years in cases and controls, respectively [5]. Yani et al. reported very low ages of 2.9 and 2.4 years in cases and controls [6]. The gender distribution in both study groups was statistically insignificant; in the case group, there were 36 females (51.4%) and 34 males (48.6%), while in the control group, there were 35 females (50%) and 35 males (50%). In Shah and Chilkar’s study, the case group had 53% males and 47% females when compared to the control group, which had 51.12% males and 48.88% females, almost similar to the present study [13]. Venturini et al.'s cases had 60% males and 40% females when compared to controls, which had 52% males and 48% females [11]. The majority of the studies had an insignificant p-value for sex distribution. But Venturini et al. and Talat et al. showed p-values less than 0.0001 and 0.003, respectively [5,11]. In our study, most of the cases were from urban areas, 58 (82.9%), but the controls were equally distributed, 35 (50%), which had statistically not similar with a p-value of 0.001. This may be a confounding factor that was not properly matched. In the study by Yani et al., among the cases, 34.3% were from rural areas and 65.7% were from the urban population [7].

In the present study, among the cases, a total of six (8.6%) had a history of TB contact, which was comparable to 11.4% in the study by Venturini et al. Shah and Chilkar reported the highest number of TB contacts at 38% [13]. In our study, 60 (85.7%) of the cases and 67 (95.7%) of the controls received the BCG vaccine, which is similar to the results of Shah and Chilkar's study, in which those percentages were 83% and 96%, respectively [13]. In Yani et al.’s study, only 42% of cases and 58% of controls were vaccinated [7]. Most of the study population (45.7%) in the current study belonged to class 4 (upper-lower) socioeconomic status according to the modified Kuppuswamy classification. Among the cases, 48.6% belonged to class 4 socioeconomic status, while in the controls, it was 42.9%. In the study of Shah and Chilkar, 79% of cases and 56% of the controls belonged to class 4 socioeconomic status [13].

In our study, among the cases, a total of 64 (91.4%) were malnourished, with 35 (50%) of them having severe malnutrition, 22 (31.4%) having moderate malnutrition, and 7 (10%) having mild malnutrition. While in the controls, 7 (10%) were mildly malnourished and four (5.7%) were moderately malnourished, with a p-value between both groups of 0.001. This means that both groups were not matched properly for malnutrition. Vitamin D levels will be lower if the nutritional status is affected. This is the major limitation of the study. The data were quite similar to the study by Shah and Chilkar [13]. The mean values were almost the same in different age groups in our study, with 16.9 ng/mL at 1-4 years of age, 17.51 ng/mL at 5-8 years of age, and 15.29 ng/mL at 9-12 years of age. Among the socioeconomic classes, lower socioeconomic class (upper lower class 4) children had the lowest vitamin D levels of 15.09 ng/mL compared to 17.16 ng/mL in the lower middle class (3) and 22.47 ng/mL in the upper middle class (2) children. Rural children have a mean vitamin D level of 21.90 ng/mL compared to that of children belonging to urban areas, with a mean value of 13.97 ng/mL. Two meta-analyses from China reported that certain VDR polymorphisms confer increased resistance to TB, while others make their hosts susceptible [14,15]. They also noted that Asians are more susceptible to TB as a result of genetic factors.

Recommendations

Randomized controlled trials are needed to evaluate the possible role of vitamin D supplementation in reducing TB disease risk. Our study suggested that improving vitamin D levels, either by exposure to sunlight or by vitamin D supplementation or food fortification, is needed for most children in this area, regardless of TB status. Further research to establish an adjuvant therapy after adjusting the proper dose to combat TB and an investigation of vitamin D mechanisms at the molecular and genetic levels are required.

Limitations

The limited number of children evaluated is the major limitation of our study. We did not assess some risk factors for TB (e.g., passive smoking) and factors influencing serum vitamin D (e.g., diet composition, food security, sunlight exposure, and VDR polymorphism). Nutrition, rural and urban areas, and BCG vaccination status were not properly matched in both groups. If 25-hydroxyvitamin D levels can be shown to have any correlation with the outcome of tubercular disease, this mode of intervention may be logical.

## Conclusions

The prevalence of VDD was high among children with TB than with controls. In addition, the severe form of VDD (<10 ng/mL) was higher among children with TB. Clinicians should be aware of associated malnutrition and low socioeconomic status as risk factors for severe VDDs among them.
